# Countergradient Variation in Reptiles: Thermal Sensitivity of Developmental and Metabolic Rates Across Locally Adapted Populations

**DOI:** 10.3389/fphys.2020.00547

**Published:** 2020-06-18

**Authors:** Amanda K. Pettersen

**Affiliations:** Department of Biology, Lund University, Lund, Sweden

**Keywords:** temperature, climate, adaptation, cogradient, incubation, embryo, maternal investment

## Abstract

Environmental temperature is a key driver of variation in developmental physiological rates in reptiles. Cooler temperatures extend development time and can increase the amount of energy required to achieve hatching success, which can pose fitness consequences later in life. Yet, for locally-adapted populations, genetic variation can oppose environmental variation across ecological gradients, known as countergradient variation (CnGV). Biologists often seek to understand the presence of phenotypic variation, yet the absence of such variation across environmental gradients can also reveal insights into the mechanisms underlying local adaptation. While evidence for genetic variation opposing environmental variation in physiological rates has been summarized in other taxa, the generality of CnGV variation in reptiles is yet unknown. Here I present a summary of studies measuring development time and metabolic rates in locally-adapted populations across thermal clines for 15 species of reptiles across 8 families. CnGV in development time is found to be common, while no clear pattern emerges for the thermal sensitivity of metabolic rates across locally-adapted populations. CnGV in development time may be an adaptive response in order to decrease the costly development in cool climates, however, empirical work is needed to disentangle plastic from genetic responses, and to uncover potentially general mechanisms of local thermal adaptation in reptiles.

## Introduction

Thermal regimes often vary considerably across spatio-temporal gradients, yet similar developmental phenotypes can be maintained when genetic variation opposes environmentally-induced variation (Levins, 1969; Conover and Schultz, 1995). Biologists have long sought to understand sources of phenotypic variation along thermal gradients, such as genotype-environment co-gradient variation (CoGV) that occur when genotypes non-randomly and positively affect phenotypes across environments (Box 1). Yet geographic variation in genotypes can also oppose environmental effects, thereby reducing, or masking observable phenotypic variation across a species thermal range (Taylor et al., 2015). This form of countergradient variation (CnGV) in thermally-sensitive traits such as physiological rates is important because it likely reflects an adaptive response, whereby selection acts to reduce phenotypic variance across environmental gradients in response to local selection regimes. Thus, investigating patterns of phenotypic uniformity in nature, rather than just phenotypic variability, can help us to understand potentially general mechanisms underlying local adaptation.

**FIGURE 1:**
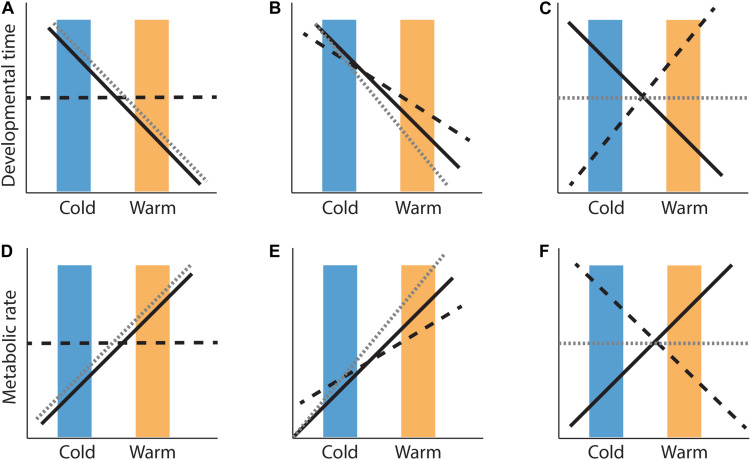
Hypothetical phenotypic variation (gray dashed lines) across locally-adapted cold (i.e., non-native conditions; blue boxes) and warm (i.e., native conditions; orange boxes) populations as a result of environmental temperature (black solid lines) and genetic (black dashed lines) effects. Shifts in phenotypic values of development time **(A–C)** and metabolic rate **(D–F)** in response to environmental temperature, can be entirely determined by environment (phenotypic plasticity; **A,D**) where the covariance between genetic (*G*) and environmental (*E*) effects = 0. Alternatively, genotypic differences can be in the same direction as environmental influences [positive Cov(*G,E*)], amplifying environmental effects on phenotypic (co-gradient variation; **B,E**), or they can oppose environmental temperature effects [negative Cov(*G,E*)], resulting in little or no phenotypic change across cold and warm environments **(C,F)**. Note, reaction norms may differ among genotypes, posing environmentally determined effects on phenotype value [V_GXE_; discussed in Box 2 in Conover and Schultz (1995), not shown here].

**Box 1. Genotype-environment covariances:**

co- and counter-gradient variation. Genotype-environment covariances [Cov(*G,E*)] can be either positive or negative, depending on whether they reinforce or oppose each other. There are three potential ways in which genotype-environment covariances can play out across populations. First, genotypes (*G*) and the environment (*E*) shift trait expression in the same direction, known as cogradient variation where the Cov(*G,E*) term is positive (e.g., Figures 1B,E). Second, trait shifts due to genotypes do not align with trait shifts due to the environment [Cov(*G,E*) is negative], referred to as countergradient adaptation (e.g., Figures 1C,F). Alternatively, phenotypes that arise from genotypes distributed randomly in a population that change only in response to the environment, are the result of phenotypic plasticity (e.g., Figures 1A,D).

There are many instances of both co- and counter- gradient variation among populations spanning a range of taxa, where natural selection drives variation across spatial and temporal thermal gradients, from microclimate and seasonal shifts, to species-level altitudinal and latitudinal ranges, or climatic shifts (Conover et al., 2009). Physiological rates often show countergradient variation: in a review by Conover et al. (2009), 87% of the studies showing evidence for CnGV were for measures of growth and developmental rates, while evidence for CoGV in physiological rates was found to be comparatively rare (Kelly, 2019). It is unclear why CnGV in the thermal sensitivity of physiological traits, relative to other traits, is so prevalent, however, it may be due to relatively weaker genetic constraints in physiological traits [compared with for example, the often strong genetic constraints of morphological traits which generally show CoGV (Li et al., 2011)]. Temperature poses a strong influence on physiological rates underlying energy acquisition and utilization in ectotherms that often misalign with the direction of selection. For example, an acute decrease in environmental temperature increases development time, yet cold climates often select for faster development so that embryos can complete development and commence feeding and growth before the onset of winter (Edge et al., 2017). CnGV can enable populations to compensate for the direct effects of temperature on physiological rates, to ensure persistence of populations under extreme climatic regimes (Angilletta, 2009; Conover et al., 2009).

In egg laying species, temperature experienced during embryonic development can impart significant fitness consequences, either through hatching success (survival) or effects imparted later in life, for example reductions in size at hatching, growth rates and reproductive success (Warner et al., 2010; Andrews and Schwarzkopf, 2012; DuRant et al., 2013; Ospina et al., 2018). Low temperatures can affect key physiological rates during development, including increasing time from fertilization to hatching (development time) and decreasing rates of energy expenditure (metabolic rate). Across a species thermal range, it is reasonable to assume both development time and metabolic rate are under stabilizing selection since adequate time and energy is needed to successfully complete cell division and differentiation, however, shifts from the optima in either trait could expose embryos to higher mortality risk via predation, desiccation, or depletion of energy reserves (Martin et al., 2007; Burton et al., 2011; Nord and Nilsson, 2011).

Combined, the thermal sensitivities of developmental and metabolic rates determine how energy use during development (fertilization until nutritional independence) scales with temperature (Pettersen et al., 2019). Increasing either development time (*D*), or metabolic rate (*MR*) will increase the costs of development, and therefore reduce the amount of residual energy at hatching. The recently proposed Development Cost Theory (DCT) posits that the relative temperature sensitivities of *D* and *MR* determine the amount of energy expended at any given temperature (Marshall et al., 2020). At cooler developmental temperatures, *D* is often increased more than *MR* decreases, hence cold environments generally increase total energy use, thereby reducing energy available for fitness-enhancing processes such as growth, maintenance and foraging (Booth and Thompson, 1991; Angilletta et al., 2000; DuRant et al., 2011; Pettersen et al., 2019), however, there are exceptions (e.g., Oufiero and Angilletta, 2010). Based on DCT, the temperature dependence of developmental rate, has the greatest influence on the relative costs of development, and is therefore expected to evolve more rapidly than the thermal sensitivity of metabolic rate (Marshall et al., 2020). DCT can thus provide a useful framework for detecting local adaptation by providing a mechanistic link between population-level reaction norms and fitness across thermal gradients.

While development time and the costs of development are generally increased at low environmental temperatures, countergradient variation can compensate for these effects. Countergradient variation can reduce the costs of development associated with cool temperatures via variation in developmental and metabolic rates that oppose the acute effects of environment on phenotype – for example, higher physiological rates can be maintained despite decreases in environment temperature. In order to identify whether the genetic component for the change in mean *D* or *MR* is statistically correlated with thermal gradient, three criteria must be met: (1) measures of the pattern of change in *D* and *MR* across a spatially or temporally varying environmental gradient; (2) the norm of reaction for *D* and *MR* in response to temperature; (3) a measure of the magnitude of thermal variation across the gradient. It is often difficult to unequivocally demonstrate that inter-populational divergence in thermal sensitivity of a trait is a result of adaptive genetic divergence, and not due to a plastic response. Obtaining evidence for CoGV and CnGV requires collecting data from common garden or reciprocal transplant studies, conducted across a range of temperatures in order to distinguish between *V*_GxE_ and Cov(*G,E*) (Yamahira and Conover, 2002; Yamahira et al., 2007). Norms of reaction that are parallel, and those that lie above or below one another in trait value provide evidence for CnGV and CoGV, respectively. Whereas, both *V*_GxE_ and Cov(*G,E*) are acting simultaneously when norms of reaction are not parallel and do not cross (see Box 2 in Conover and Schultz, 1995). While the prevalence of CoGV and CnGV in physiological traits has been reviewed in fish (Conover et al., 2006), amphibians (Morrison and Hero, 2003), marine invertebrates (Sanford and Kelly, 2011), and insects (Sinclair et al., 2012), examples in reptiles are less well documented. This is surprising, given that reptiles represent one of the largest study groups in vertebrate thermal physiology.

It is important to develop a clear understanding of patterns of countergradient variation in nature, before designing experiments to evaluate causal mechanisms (Conover et al., 2009). This paper therefore compiles data from common garden (CG) and reciprocal transplant (RT) studies testing for temperature-by-population interactions on variations in development time (*D*) and metabolic rate (*MR*) across cold- and warm-adapted populations of reptiles (Li et al., 2018a). Effect sizes for each study, weighted by sample size can then be calculated in order to test whether selection has modified reaction norms of *D* and *MR* across climatic regimes (Supplementary Table S1). It is anticipated that despite a decrease in environmental temperatures, cold-adapted populations maintain similar *D* and (or) *MR* across a species’ thermal gradient, relative to warm-adapted populations. Reptiles provide a useful model system to study local adaptation because developmental trajectories in reptiles are highly sensitive to environmental temperatures (Angilletta, 2009), and many reptile species have limited dispersal ability between populations (Uller and While, 2015). This review aims to elucidate broad-scale mechanisms underlying local adaptation in reptiles by evaluating the generality of phenotypic plasticity [Cov(*G,E*) = 0; Figures 1A,D], cogradient variation [Cov(*G,E*) > 1; Figures 1B,E], and countergradient variation [Cov(*G,E*) < 1; Figures 1C,F] in developmental physiological rates across populations experiencing different thermal regimes. If populations maintain similarity in *D* and (or) *MR* under thermal change then evolution is likely the result of CnGV, whereas rapid trait divergence in *D* or *MR* in the direction of thermal change is due to the evolution of CoGV. Due to the paucity of data on thermal sensitivity of *D* and *MR*, it is not yet feasible to present a formal, comprehensive meta-analysis on the topic here. Rather, this review serves as a summary of existing data on thermal reaction norms across locally adapted populations, and points toward future avenues of research that require further work in order to continue developing our understanding of adaptation along thermal gradients.

### Countergradient Variation of Thermal Sensitivity in Reptiles Is Prevalent in Developmental but Not Metabolic Rates

**FIGURE 2:**
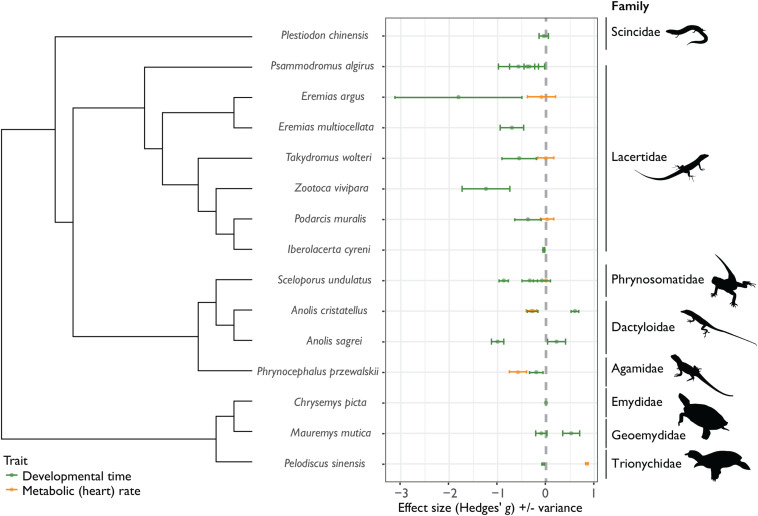
Effect sizes (Hedges’ *g*) for differences in the thermal sensitivity of development time (time from oviposition until hatching) and metabolic (heart) rate across cold and warm-adapted populations for 15 species of reptiles across 8 families (± variance). For development time (*D*; green data points and variance bars), positive Hedges’ *g* values indicate positive Cov(*G,E*), or cogradient variation, where cold-adapted populations have longer *D*, relative to warm-adapted populations. Negative values of *D* indicate negative Cov(*G,E*), or countergradient variation, where genotypic differences oppose environmental temperature effects – in these instances, cold-adapted populations develop faster than warm-adapted populations. For metabolic rates (*MR*; orange data points and variance bars), negative Hedges’ *g* values indicate positive Cov(*G,E*), or cogradient variation, where cold-adapted populations have lower *MR*, relative to warm-adapted populations, while positive values of *MR* indicate negative Cov(*G,E*), or countergradient variation – here cold-adapted populations maintain higher *MR*, relative to warm-adapted populations.

Most published studies show evidence for CnGV between development time and environmental temperature [negative values of Hedges’ *g* (*D*); Figure 2], supporting the generality of countergradient variation in reptile development. For 17 out of 22 studies, intrinsic (genetic) factors were shown to counter thermal influences on developmental rate. Rather than an outcome of genetic drift, these findings suggest an adaptive countergradient response – selection opposes reaction norms of development time (*D*) across climatic regimes. Development under cool conditions necessitates a countergradient adaptive response for faster development and earlier hatching time, enabling embryos to hatch before winter while resources are still available (Du et al., 2012). On the contrary, there is little support to suggest that CnGV is common for metabolic rate (*MR*) – overall, reptile embryos from locally-adapted cooler climates did not maintain higher metabolic rates compared with populations from warmer climates (Figure 2). This could be due to several reasons, not least that metabolic rate is a highly variable trait, even after correcting for mass and temperature (Burton et al., 2011). There is also evidence for population-level differences in thermal reaction norms in heart rate across development stage (Angilletta et al., 2013). Since metabolic rate is not a single trait (Pettersen et al., 2018), multiple measures of *MR* throughout development are needed in order to elucidate patterns in rates of energy expenditure across locally-adapted populations.

Despite an apparent lack of evolved response in MR to buffer against reduced energy turnover under cold temperatures, a countergradient response in *D* can itself reduce energy expenditure during development under cold conditions. Across a species’ natural temperature range, the thermal sensitivity of *D* is often greater than the thermal sensitivity of *MR* and is therefore a more important determinant of how the costs of development scale with temperature (Pettersen et al., 2019; Marshall et al., 2020). It may be that embryos counteract increased energy costs associated with development under cold temperatures, by reducing *D* without a concomitant increase in *MR*, and is supported by evidence for CnGV in yolk assimilation in the eastern fence lizard (Storm and Angilletta, 2007). The ability to evolve increases in one physiological rate independently of another has been shown previously for metabolic and growth rates (Williams et al., 2016). It may be that for developmental physiological rates, selection acts to reduce the costs of development, via CnGV in the thermal sensitivity of *D*, resulting in a closer alignment of embryo thermal optima to that of local thermal regimes.

### Proximal Drivers of Countergradient Adaptation in Developmental Rates

Various mechanistic explanations have been proposed to explain the prevalence of countergradient variation in developmental rates, and its compensating effects on the costs of development at cooler temperatures, including the role of maternal effects. It is a common view that faster development in cold-, vs. warm-adapted populations reared under common garden conditions are a consequence of later stage of embryogenesis at laying, earlier stage of development at hatching, or larger egg size. For example, extended embryo retention and greater maternal provisioning in order to reduce *D* is often associated with cool climates in squamate reptiles, both among and within species (Shine, 1995; While et al., 2015). Yet, even after accounting for population-level differences in maternal investment, studies find faster developmental rates in cold-adapted populations (Storm and Angilletta, 2007; Du et al., 2010). Increased heart mass in cold-adapted populations may allow for faster cell division and differentiation associated with development (Du et al., 2010), however, how these effects are isolated from effects on the thermal sensitivity of heart rates is difficult to explain. Variation in yolk hormone content and composition (Ruuskanen et al., 2016) or enzymatic activity (Rungruangsak-Torrissen et al., 1998) may also play a role in facilitating faster developmental rates in cold-, relative to warm-adapted populations. Indeed, there are likely to be multiple mechanisms responsible for countergradient variation in *D*, rather than any single factor.

### Consequences of Countergradient Adaptation: When and Why Is Thermal Countergradient Adaptation Absent?

Despite the prevalence of CnGV in development time, there are studies that do not show this trend, for example evidence for CnGV was absent across native-non-native ranges for species adapting to hot temperatures. When comparing forested (cool) vs. urban (hot) populations of *Anolis cristatellus* and *A. sagrei* under common garden conditions, co-gradient variation was observed where hot-adapted populations showed shorter development times relative to warm-adapted populations (Tiatragul et al., 2017; Hall and Warner, 2018). These findings are congruent with CDT – beyond a species usual temperature range development is more costly because metabolic rate increases more than development time decreases (Marshall et al., 2020). Decreasing *D* at hot temperatures results in reduced costs of development, and therefore likely fitness advantages. Further measures of the relative temperature dependencies of *D* and *MR* in other species are needed to elucidate the temperature-dependent costs of development as a potentially general mechanism for local thermal adaptation to extreme high temperatures.

Trade-offs with other fitness-related traits can also help to explain an absence of CnGV in *D* – reducing development time may come at the cost of embryos hatching at smaller sizes and reduced juvenile growth rate (Angilletta et al., 2003; Buckley et al., 2010). However, in reptiles, evidence for trade-offs amongst life-history traits are mixed (Niewiarowski and Angilletta, 2008; Fetters and McGlothlin, 2017). Alternatively, it may be that selection on other traits can compensate for a lack of CnGV in developmental physiological rates. In squamates, behavioral thermoregulation, such as shifts in female body temperature while gravid, may be a more labile, and therefore more important mechanism for adaptation to cold and variable climatic regimes than perhaps more conserved, physiological responses (Navas, 2002).

Other climatic factors that vary across thermal gradients, such as temperature variation, seasonal time constraints, and food availability may confound effects of temperature on developmental rates. Studies using fluctuating, rather constant temperature manipulations showed mixed results, such as the absence population-level patterns (Angilletta et al., 2013) or evidence for CoGV (Li et al., 2018b). Both CoGV and CnGV were found under temperatures that were fluctuating, but representative of natural nest temperatures (Oufiero and Angilletta, 2006; Li et al., 2018b; Figure 2 and Supplementary Table S1). In order to capture realistic, population-level responses, it is important that temperature manipulations reflect natural thermal conditions that can account for non-linearity in the thermal dependence of physiological rates (Denny, 2017). Further, thermal fluctuation studies offer complex, albeit vital insights into whether CnGV can be maintained under rapid environmental change, and more studies are needed to reveal any consistent patterns across magnitudes of spatial and temporal thermal gradients (Du et al., 2010; Li et al., 2018a).

Finally, it may be that interactions between genotype and environment are inflating observations of CnGV across thermal regimes, and that CnGV in development time is less common than currently assumed. There may be genotype-dependent effects of environment on development time, where for example, a single genotype is superior in all environments, even though the slopes of reaction norms differ (see Box 2 in Conover and Schultz, 1995). All studies included in this review only observed a single generation – it is unlikely that all sources of *V*_*E*_ are controlled for over this timescale (plastic responses may still play a role), which is a limitation of studying species with relatively long generation times, such as reptiles and other vertebrates (Laugen et al., 2003).

### Future Directions for Understanding Local Adaptation via Evolution of Developmental Rates in Reptiles

Adaptation of developmental physiological rates is an important, yet underutilized avenue of research for understanding population persistence under changing and novel environments. Countergradient variation for traits expressed later in the life history have been well documented in reptiles, including growth (Sears and Angilletta, 2003; Uller and Olsson, 2003; Li et al., 2011; Snover et al., 2015; Ortega et al., 2017), body size (Oufiero et al., 2011; Iraeta et al., 2013), scale size (Oufiero et al., 2011), preferred body temperature (Hodgson and Schwanz, 2019), nest date (Knapp et al., 2006; Edge et al., 2017), reproductive output (Knapp et al., 2006; Li et al., 2011; Fetters and McGlothlin, 2017), critical thermal limits and water loss (Kolbe et al., 2014) and locomotor performance (Niewiarowski, 2001; McElroy, 2014). It is possible that CnGV for traits observed later in life are also a consequence of developmental environment, such as food availability, temperature and stress (DuRant et al., 2013; Noble et al., 2018).

Developmental and metabolic rates under selection may in turn affect selection on genetically correlated traits later in life (Artacho et al., 2015; Pettersen et al., 2016, 2018; Ricklefs et al., 2017). Resolving the interplay between plastic and genetic responses to local selective forces throughout the life history and environmental gradients is the next fundamental challenge (Buckley et al., 2010). Isolating the role of maternal effects from environmental effects can be challenging without multi-generational studies, nevertheless, investigating the effect of maternal environment on offspring phenotype can provide insight into mechanisms underlying rapid adaptation to novel environments. Variation in maternal investment along environmental gradients is common in reptiles, even in egg-laying species that buffer their offspring from external temperatures via behavioral (Mathies and Andrews, 1996; Du et al., 2010), physiological (Harlow and Grigg, 1984) or endocrinological (Uller et al., 2007) mechanisms which may complement or even drive countergradient variation to facilitate acclimation and adaptation to local thermal regimes.

## Conclusion

Across latitudinal and altitudinal clines, cold-adapted populations have genetic capacity for faster development, relative to warm-adapted populations. While these differences in thermal sensitivity to local temperatures did not extend to warm- vs. hot-adapted populations such as forested vs. city populations, there is overall support for common CnGV in development time in reptiles, which mirrors findings observed in other taxa (Conover et al., 2009). Given the highly sensitive nature of developmental trajectories to acute changes in temperature, maintenance of stable physiological rates in species covering wide distributions offers a fascinating avenue for understanding local adaptation (Du et al., 2010). In particular, evolutionary change in the thermal sensitivity of developmental and metabolic rates is likely to be a crucial component of adaptive responses to environmental change (Kelly, 2019). Identifying the nature of genotype-environment covariances across ecological gradients is key to understanding variation in physiological rates and for predicting population persistence under environmental change.

## Author Contributions

AP conceived of the idea, collected the data, and wrote the manuscript.

## Conflict of Interest

The author declares that the research was conducted in the absence of any commercial or financial relationships that could be construed as a potential conflict of interest.
