# Corrigendum: Countergradient Variation in Reptiles: Thermal Sensitivity of Developmental and Metabolic Rates Across Locally Adapted Populations

**DOI:** 10.3389/fphys.2021.652269

**Published:** 2021-02-18

**Authors:** Amanda K. Pettersen

**Affiliations:** Department of Biology, Lund University, Lund, Sweden

**Keywords:** temperature, climate, adaptation, cogradient, incubation, embryo, maternal investment

In the original article, there was a misinterpretation of the results of Tiatragul et al., 2017 and Hall and Warner, 2018, where population-level differences in development time for *A. cristatellus* and *A. sagrei* were incorrectly stated.

These studies did not find any significant effect of habitat of origin on development time – mean incubation times between forested and urban wild populations were similar across temperature treatments. Tiatragul et al. (2017) showed slight differences in incubation duration between forested and urban populations (Figure 2), however these were not significant (Table 1). The “Forest” and “City” headings of Table 1 in Hall and Warner (2018) refer to the incubation treatments (forest or city thermal profile), not the population – since no population x incubation treatment interactions were found, data across populations were pooled to estimate mean incubation period for each treatment.

**Figure 2:**
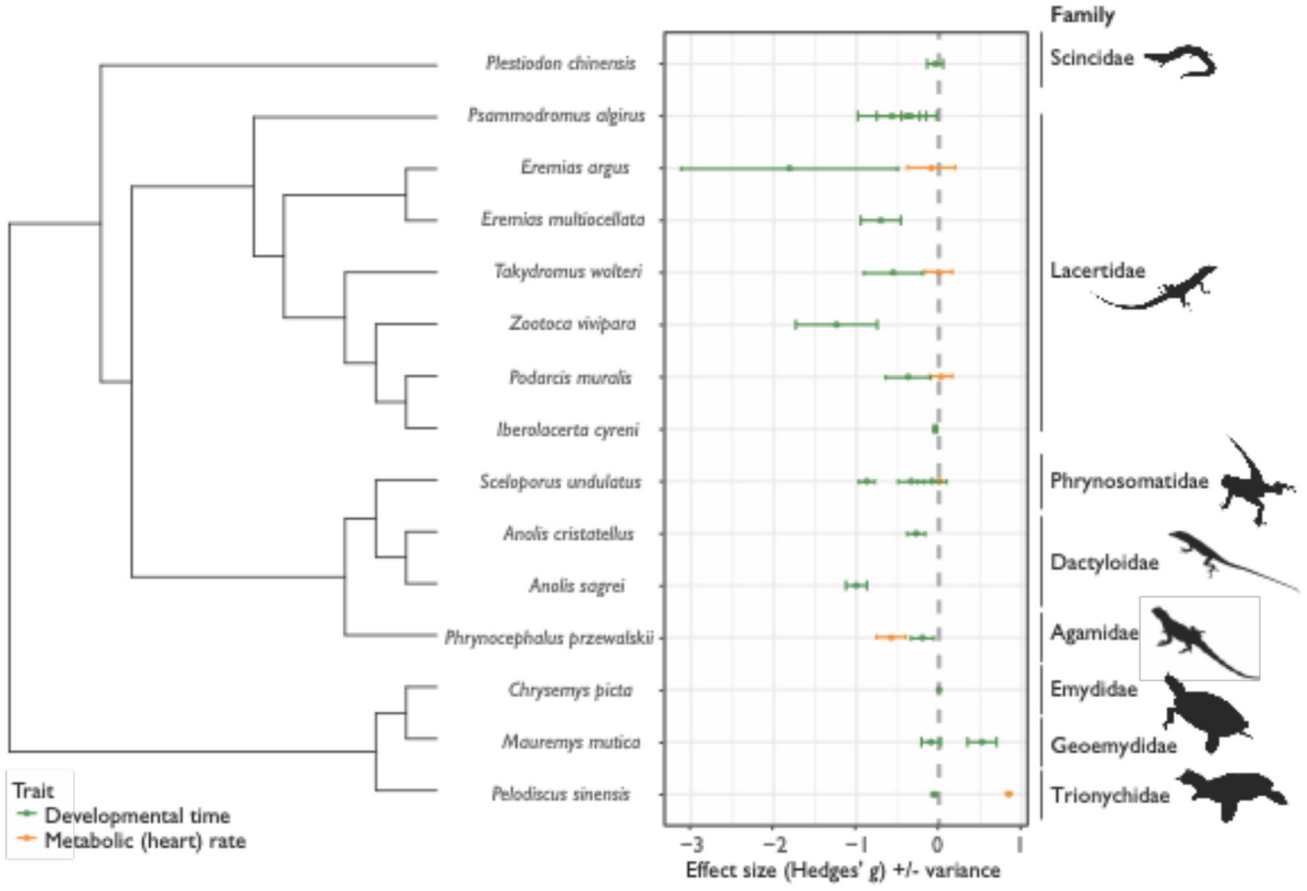
Effect sizes (Hedges' *g*) for differences in the thermal sensitivity of development time (time from oviposition until hatching) and metabolic (heart) rate across cold and warm-adapted populations for 15 species of reptiles across 8 families (± variance). For development time (*D*; green data points and variance bars), positive Hedges' *g* values indicate positive Cov(*G,E*), or cogradient variation, where cold-adapted populations have longer *D*, relative to warm-adapted populations. Negative values of *D* indicate negative Cov(*G,E*), or countergradient variation, where genotypic differences oppose environmental temperature effects – in these instances, cold-adapted populations develop faster than warm-adapted populations. For metabolic rates (*MR*; orange data points and variance bars), negative Hedges' *g* values indicate positive Cov(*G,E*), or cogradient variation, where cold-adapted populations have lower *MR*, relative to warm-adapted populations, while positive values of *MR* indicate negative Cov(*G,E*), or countergradient variation – here cold-adapted populations maintain higher *MR*, relative to warm-adapted populations.

A correction has been made to **“*Consequences of Countergradient Adaptation: When and Why Is Thermal Countergradient Adaptation Absent?”:***

Despite the prevalence of CnGV in development time, there are studies that do not show this trend, for example evidence for CnGV was absent across native-non-native ranges for species adapting to hot temperatures. Differences in development time were absent when comparing forested (cool) vs. urban (hot) populations of *Anolis cristatellus* and *Anolis sagrei* under common garden conditions (Tiatragul et al., 2017; Hall and Warner, 2018). Further measures of the relative temperature dependencies of *D* and *MR* in other species are needed to elucidate the temperature-dependent costs of development as a potentially general mechanism for local thermal adaptation to extreme high temperatures.

In the original article, there was a mistake in Figure 2 as published. Due to the misinterpretation of results by Hall and Warner, 2018 (as per above), effect sizes for development time of *Anolis cristatellus* were incorrect. Since data across populations were pooled for this study, effect sizes were unable to be recalculated. The corrected Figure 2 appears below.

The authors apologize for this error and state that this does not change the scientific conclusions of the article in any way. The original article has been updated.
