# Supplementary material for: Countergradient Variation in Reptiles: Thermal Sensitivity of Developmental and Metabolic Rates Across Locally Adapted Populations
Source: Front Physiol. 2020 Jun 18;11:547. doi: 10.3389/fphys.2020.00547 (PMC7314978; doi:10.3389/fphys.2020.00547)
Supplement: Supplementary file 1 [file Table_1.pdf]

Supplementary Material

Title: Countergradient variation in reptiles: thermal sensitivity of developmental and metabolic rates across locally adapted populations

Author affiliation: Amanda K. Pettersen[1*]

[1] Department of Biology, Lund University, Sölvegatan 37, 22362, Lund, Sweden

*Corresponding author: Email: amanda.pettersen@biol.lu.se

Methods

Meta-analysis for thermal sensitivity of developmental and metabolic rates across locally adapted populations

Published articles presenting data on developmental and metabolic rates across populations of reptiles were collected from ISI *Web of Science* using search terms "local adaptation", "latitude", "altitude" OR "gradient" AND "development*", AND "temperat*" OR "therm*" together with the terms "lizard" OR "snake" OR "reptile". This search yielded 83 unique entries, and of these 62 were rejected based on irrelevance. Studies were only included if they included either a common garden (CG) or reciprocal transplant (RT) method, whereby reptile species from more than one population were collected and eggs incubated under either a single (CG) or multiple (RT) ecologically relevant, experimentally controlled, temperatures. Development time was measured as the number of hours from oviposition until hatching (h). Metabolic rate during development was measured as embryo heart rate in beats per minute (BPM). Studies were only included if they reported sample size or a test statistic from which sample size could be calculated (e.g., degrees of freedom) as well as a measure of error from which standard deviation could be determined in order to minimise sampling bias.

I calculated effect sizes for each study using Hedges' *g*, which provides unbiased, standardised mean differences in *D* or *MR* for comparisons between cold- and warm-adapted populations, while adjusting effects for small sample sizes (Borenstein et al., 2009). To account for shared evolutionary history among species driving any observed patterns in *D* and *MR* reaction norms, I constructed a tree topology using the open tree of life and the 'rotl' package and compared the fit of models with or without phylogeny included using the 'pgls' function in the R package 'caper' and Rv3.6.1 (Orme et al., 2013; Hinchliff et al., 2015; Michonneau et al., 2016) . When comparing models with ("phylogeny model") and without ("null model") phylogeny, I found that phylogeny did not explain sufficient variance in

Hedges' *g*, ($AIC_{phylogeny}$: 94.42 > AIC_{null}: 83.52), hence effect sizes were not corrected for phylogenetic signal.

Publication bias

To test for publication bias (arising from sampling a greater proportion of statistically significant, and therefore published results) I explored the relationship between the effect size, (Hedges' *g*) and precision of each estimate (1/SE) using funnel plots (Borenstein *et al.* 2011). In the case of publication bias, we would expect to see an absence of effect sizes from studies with small sample size, and therefore low precision. Despite a paucity of available studies meeting the meta-analysis criteria, effect sizes were distributed symmetrically around the mean, and there appeared to be little evidence of publication bias.

Table S1: Summary of reptile data used to calculate effect sizes (Hedges' *g*) for differences in the thermal sensitivity of developmental time (D) and metabolic (heart) rate (MR) across cold and warm-adapted populations. Data includes studies reporting development time (D; time from oviposition until hatching) and metabolic rate (HR; measured as heart rate) for embryos measured across either a single incubation temperature (common garden; CG) or multiple incubation temperatures (reciprocal transplant; RT). Hedges' *g* provides standardised mean differences in D and HR between cold- and warm-adapted populations that adjusts effects for small sample sizes. Positive Hedges' *g* values indicate cold-adapted populations have longer developmental times (D) or higher metabolic rates (HR) relative to warm-adapted populations (and vice versa), NS (non-significant). Species natural nest temperature ranges (ºC) are included where provided by each study, and incubation temperature variance (ºC) is provided where studies used fluctuating thermal regimes.

Family	Species	Trait	Gradient	Method	Natural nest temperature range (ºC)	Incubation temperatures (ºC) and variance	Effect size (Hedges' *g*)	Evidence for CoGV or CnGV?	Reference
	Order: Squamata								
Agamidae	*Phrynocephalus przewalskii*	DT	Climate (cold/warm)	CG	N/A	28.0	-0.201	CnGV	Zeng et al., (2013)
Agamidae	*Phrynocephalus przewalskii*	MR	Climate (cold/warm)	CG	N/A	28.0	-0.579	CoGV	Zeng et al., (2013)
Dactyloidae	*Anolis cristatellus*	DT	Temperature (cold/warm)	CG	25.5 – 33.6	26.7	-0.276	CnGV	Tiatragul et al., (2017)
Dactyloidae	*Anolis cristatellus*	DT	Climate (cold/warm)	RT	25 – 43	26.5, 29.0, 39, 43	0.597	CoGV	Hall and Warner (2018)

Dactyloidae	*Anolis cristatellus*	MR	Climate (cold/warm)	RT	25 – 43	26.5, 29.0, 39, 43	-0.302	CoGV	Hall and Warner (2018)
Dactyloidae	*Anolis sagrei*	DT	Temperature (cold/warm)	CG	25.5 – 33.6	26.7	0.218	CoGV	Tiatragul et al., (2017)
Dactyloidae	*Anolis sagrei*	DT	Latitude (cold/warm)	CG	N/A	28.0	-0.100	CnGV	Fetters and McGlothlin (2017)
Lacertidae	*Eremias multiocellata*	DT	Altitude (cold/warm)	RT	N/A	25.0 (7), 29.0 (11), 31.0 (13), 35.0 (17)	-0.705	CnGV	Tang et al., (2012)
Lacertidae	*Eremias argus*	DT	Altitude (cold/warm)	CG	15.5 – 31.8	24.0 (6)	-1.810	CnGV	Sun et al., (2013)
Lacertidae	*Eremias argus*	MR	Altitude (cold/warm)	RT	15.5 – 31.8	24.0 (6), 28.0 (6)	-0.093	CoGV	Sun et al., (2013)
Lacertidae	*Iberolacerta cyreni*	DT	Altitude (cold/warm)	RT	N/A	22.0, 26.0, 30.0	-0.045	NS	Monasterio et al., (2016)
Lacertidae	*Podarcis muralis*	DT	Latitude (cold/warm)	RT	9.5 – 33.5	20.0, 24.0	-0.373	CnGV	While et al., (2015)
Lacertidae	*Podarcis muralis*	MR	Latitude (cold/warm)	RT	9.5 – 33.5	20.0, 24.0	0.023	NS	While et al., (2015)
Lacertidae	*Psammodromus algirus*	DT	Altitude, Latitude (cold/warm)	CG	N/A	28.0 (4)	-0.344	CnGV	Díaz et al., (2012)
Lacertidae	*Psammodromus algirus*	DT	Altitude (cold/warm)	CG	N/A	24.0	-0.569	CnGV	Verdú-Ricoy et al., (2014)
Lacertidae	*Psammodromus algirus*	DT	Altitude (cold/warm)	RT	N/A	27.0, 30.0	-0.391	CnGV	Iraeta et al., (2006)
Lacertidae	*Takydromus wolteri*	DT	Altitude (cold/warm)	CG	15.5 – 31.8	24.0	-0.555	CnGV	Sun et al., (2013)
Lacertidae	*Takydromus wolteri*	MR	Altitude (cold/warm)	RT	15.5 – 31.8	24.0, 28.0	-0.004	NS	Sun et al., (2013)

Lacertidae	*Zootoca vivipara*	DT	Altitude (cold/warm)	RT	1 – 35	21.0, 25.0, 29.0	-1.241	CnGV	Rodriquez-Diaz and Braña (2012)
Phrynosomatidae	*Sceloporus undulatus*	DT	Latitude (cold/warm)	RT	20 – 34	24.0 (10), 27.0 (14)	-0.874	CnGV	Oufiero and Angilletta Jr (2006)
Phrynosomatidae	*Sceloporus undulatus*	DT	Altitude, Latitude (cold/warm)	CG	20 – 34	27.0 (14)	-0.084	NS	Niewiarowski and Angilletta Jr (2008)
Phrynosomatidae	*Sceloporus undulatus*	DT	Latitude (cold/warm)	RT	20 – 33.5	25.0, 28.0	-0.336	CnGV	Du et al., (2010)
Phrynosomatidae	*Sceloporus undulatus*	MR	Climate (cold/warm)	RT	20 – 33.5	25.0, 28.0	0	No	Du et al., (2010)
Scincidae	*Plestiodon chinensis*	DT	Altitude (cold/warm)	RT	25 – 31	24.0, 28.0, 32.0	-0.047	NS	Lu et al., (2014)
	Order: Testudines								
Emydidae	*Chrysemys picta*	DT	Latitude (cold/warm)	RT	N/A	27.5, 28.5	0	No	Bodensteiner et al., (2019)
Geoemydidae	*Mauremys mutica*	DT	Latitude (cold/warm)	RT	21.5 – 29.2	26.0, 28.0, 30.0	-0.097	CnGV	Zhao et al., (2015)
Geoemydidae	*Mauremys mutica*	DT	Latitude (cold/warm)	RT	23.2 – 29.5	26.0 (3), 28.0 (1.5)	0.520	CoGV	Li et al., (2018)
Trionychidae	*Pelodiscus sinensis*	DT	Latitude (cold/warm)	RT	18.6 – 37.2	26.0 (3), 31.0 (3)	-0.059	CnGV	Li et al., (2018)
Trionychidae	*Pelodiscus sinensis*	MR	Latitude (cold/warm)	RT	18.6 – 37.2	26.0 (3), 31.0 (3)	0.844	CnGV	Li et al., (2018)

<u>References</u>

Bodensteiner, B. L., Warner, D. A., Iverson, J. B., Milne-Zelman, C. L., Mitchell, T. S., Refsnider, J. M., et al. (2019). Geographic variation in thermal sensitivity of early life traits in a widespread reptile. *Ecology and Evolution* 9, 2791–2802. doi:10.1002/ece3.4956.

Borenstein, M., Hedges, L. V., Higgins, J. P., and Rothstein, H. R. (2009). “Effect Sizes Based on Means,” in *Introduction to Meta-Analysis* (Chichester, UK: John Wiley & Sons, Ltd), 21–32. doi:10.1002/9780470743386.ch4.

Díaz, J. A., Iraeta, P., Verdú-Ricoy, J., Siliceo, I., and Salvador, A. (2012). Intraspecific Variation of Reproductive Traits in a Mediterranean Lizard: Clutch, Population, and Lineage Effects. *Evol Biol* 39, 106–115. doi:10.1007/s11692-011-9144-5.

Du, W., Warner, D. A., Langkilde, T., Robbins, T., Shine, R., and Benkman, N. H. E. C. W. (2010). The Physiological Basis of Geographic Variation in Rates of Embryonic Development within a Widespread Lizard Species. *The American Naturalist* 176, 522–528. doi:10.1086/656270.

Fetters, T. L., and McGlothlin, J. W. (2017). Life histories and invasions: accelerated laying rate and incubation time in an invasive lizard, Anolis sagrei. *Biol J Linn Soc* 122, 635–642. doi:10.1093/biolinnean/blx102.

Hall, J. M., and Warner, D. A. (2018). Thermal spikes from the urban heat island increase mortality and alter physiology of lizard embryos. *Journal of Experimental Biology* 221. doi:10.1242/jeb.181552.

Hinchliff, C. E., Smith, S. A., Allman, J. F., Burleigh, J. G., Chaudhary, R., Coghill, L. M., et al. (2015). Synthesis of phylogeny and taxonomy into a comprehensive tree of life. *PNAS* 112, 12764–12769. doi:10.1073/pnas.1423041112.

Iraeta, P., Monasterio, C., Salvador, A., and Diaz, J. A. (2006). Mediterranean hatchling lizards grow faster at higher altitude: a reciprocal transplant experiment. *Functional Ecology* 20, 865–872. doi:10.1111/j.1365-2435.2006.01162.x.

Li, T., Cao, P., Bei, Y.-J., and Du, W.-G. (2018). Latitudinal and Temperature-Dependent Variation in Embryonic Development Rate and Offspring Performance in a Freshwater Turtle. *Physiological and Biochemical Zoology* 91, 673–681. doi:10.1086/694856.

Lu, H.-L., Lin, Z.-H., Li, H., and Ji, X. (2014). Geographic variation in hatchling size in an oviparous skink: effects of maternal investment and incubation thermal environment. *Biological Journal of the Linnean Society* 113, 283–296. doi:10.1111/bij.12322.

Michonneau, F., Brown, J. W., and Winter, D. J. (2016). rotl: an R package to interact with the Open Tree of Life data. *Methods in Ecology and Evolution* 7, 1476–1481. doi:10.1111/2041-210X.12593.

Monasterio, C., Verdu-Ricoy, J., Salvador, A., and Diaz, J. A. (2016). Living at the edge: lower success of eggs and hatchlings at lower elevation may shape range limits in an

alpine lizard. *Biological Journal of the Linnean Society* 118, 829–841. doi:10.1111/bij.12766.

Niewiarowski, P. H., and Angilletta Jr, M. J. (2008). Countergradient variation in embryonic growth and development: do embryonic and juvenile performances trade off? *Functional Ecology* 22, 895–901. doi:10.1111/j.1365-2435.2008.01441.x.

Orme, D., Freckleton, R. P., Thomas, G. H., Petzoldt, T., Fritz, S. A., and Isaac, N. (2013). CAPER: comparative analyses of phylogenetics and evolution in R. *Methods in Ecology and Evolution* 3, 145–151.

Oufiero, C. E., and Angilletta, M. J. (2006). Convergent evolution of embryonic growth and development in the eastern fence lizard (Sceloporus undulatus). *Evolution* 60, 1066–1075.

Rodriguez-Diaz, T., and Brana, F. (2012). Altitudinal variation in egg retention and rates of embryonic development in oviparous Zootoca vivipara fits predictions from the cold-climate model on the evolution of viviparity. *Journal of Evolutionary Biology* 25, 1877–1887. doi:10.1111/j.1420-9101.2012.02575.x.

Sun, B.-J., Li, S.-R., Xu, X.-F., Zhao, W.-G., Luo, L.-G., Ji, X., et al. (2013). Different mechanisms lead to convergence of reproductive strategies in two lacertid lizards (Takydromus wolteri and Eremias argus). *Oecologia* 172, 645–652. doi:10.1007/s00442-012-2524-4.

Tang, X.-L., Yue, F., Yan, X.-F., Zhang, D.-J., Xin, Y., Wang, C., et al. (2012). Effects of gestation temperature on offspring sex and maternal reproduction in a viviparous lizard (Eremias multiocellata) living at high altitude. *Journal of Thermal Biology* 37, 438–444. doi:10.1016/j.jtherbio.2012.03.002.

Tiatragul, S., Kurniawan, A., Kolbe, J. J., and Warner, D. A. (2017). Embryos of non-native anoles are robust to urban thermal environments. *Journal of Thermal Biology* 65, 119–124. doi:10.1016/j.jtherbio.2017.02.021.

Verdú-Ricoy, J., Iraeta, P., Salvador, A., and Díaz, J. A. (2014). Phenotypic responses to incubation conditions in ecologically distinct populations of a lacertid lizard: a tale of two phylogeographic lineages. *Journal of Zoology* 292, 184–191. doi:10.1111/jzo.12091.

While, G. M., Williamson, J., Prescott, G., Horvathova, T., Fresnillo, B., Beeton, N. J., et al. (2015). Adaptive responses to cool climate promotes persistence of a non-native lizard. *Proc. R. Soc. B-Biol. Sci.* 282, 20142638. doi:10.1098/rspb.2014.2638.

Zeng, Z.-G., Zhao, J.-M., and Sun, B.-J. (2013). Life history variation among geographically close populations of the toad-headed lizard (Phrynocephalus przewalskii): Exploring environmental and physiological associations. *Acta Oecologica* 51, 28–33. doi:10.1016/j.actao.2013.05.004.

Zhao, B., Chen, Y., Lu, H.-L., Zeng, Z.-G., and Du, W.-G. (2015). Latitudinal differences in temperature effects on the embryonic development and hatchling phenotypes of the Asian yellow pond turtle, Mauremys mutica. *Biological Journal of the Linnean Society* 114, 35–43. doi:10.1111/bij.12400.
